# Oral lichenoid lesion simultaneously associated with Castleman’s disease and papillary thyroid carcinoma: a rare case report

**DOI:** 10.1186/s12903-022-02623-2

**Published:** 2022-12-07

**Authors:** Jiaying Zhou, Rui Zhou, Pingping Tan, Bin Cheng, Liwei Ma, Tong Wu

**Affiliations:** 1 Hospital of Stomatology, Guanghua School of Stomatology, Sun Yat-Sen University, 56 Lingyuan Road West, Guangzhou, 510055 Guangdong China; 2 Guangdong Provincial Key Laboratory of Stomatology, Guangzhou, 510080 China; 3 Department of Pathology, Hunan Cancer Hospital, Changsha, 410031 Hunan China; 4 Department of Oral Medicine, Center of Stomatology, Xiangya Hospital, Central South University, Changsha, 410008 Hunan China; 5 Institute of Oral Cancer and Precancerous Lesions, Central South University, Changsha, 410008 Hunan China; 6 National Clinical Research Center for Geriatric Disorders, Xiangya Hospital, Central South University, Changsha, 410008 Hunan China

**Keywords:** Oral lichenoid lesion, Castleman’s disease, Papillary thyroid carcinoma, Case report

## Abstract

**Background:**

Oral lichenoid lesion (OLL) is a term used to describe oral lesions that have clinical and/or histopathological features similar to oral lichen planus (OLP), but it is thought to be caused by specific triggers or systemic conditions and presents higher malignant transformation rate than OLP. To date, OLL simultaneously complicated with Castleman’s disease (CD) and papillary thyroid carcinoma (PTC) has not been reported. Reporting from such disorders is crucial to avoid misdiagnosis and help in timely intervention.

**Case presentation:**

We report a rare case of a 39-year-old female with extensive ulcerated lesions on the oral mucosa, diagnosed as OLL by histopathology. Routine oral treatment was scheduled to control the OLL, while the oral lesions remained unhealed. Computed tomography examination was performed after the oral treatment and revealed thyroid and mediastinal masses, which were then surgically removed and pathologically diagnosed as PTC and CD, respectively. Two months after complete excision of the neoplasms, the oral lesions showed obvious alleviation. With subsequent treatment for oral lesions, the patient’s OLL healed.

**Conclusions:**

This is the first reported OLL case simultaneously associated with CD and PTC. This case reminds us to focus on the underlying etiologies of OLL and the multidisciplinary collaboration for oral lesions associated with systemic diseases.

## Background

Oral lichenoid lesion (OLL) is a chronic inflammatory disease of unknown etiologies or pathogenesis with varied disease severity, which appears as dotted grey-white streaks, reticulated and plaque-like keratotic papules, accompanied by mucosal congestion, erosion, ulceration, atrophy and blistering [1, 2]. The etiologies of OLL are probably associated with exogenous triggers such as dental restorative materials and systemic medications, and it can also be induced by systemic diseases [3]. The disparities on the etiologies may profoundly influence the treatment strategies [4, 5].

OLL and oral lichen planus (OLP) share several clinical and pathological characteristics as well as oral treatment regimens [2]. The hyperkeratotic striation/reticulation, varying degrees of erosions/ulceration and histopathological inflammatory changes are considered suggestive of OLL and OLP [6]. However, OLL is thought to be caused by specific triggers or systemic conditions, and exhibits more extensive and deeper inflammatory infiltration of lymphocytes, and more eosinophils, plasma cells and granulocytes [7]. Moreover, with respect to the clinical prognosis, OLL has increased risk of malignant transformation than OLP [8, 9]. Herein, scrutinizing potential etiologies, making an accurate diagnosis and developing an optimal treatment plan for OLL are essential for better prognosis.

Castleman’s disease (CD) is a rare heterogeneous lymphoproliferative disorder, and occasionally co-occurs with oral changes such as paraneoplastic pemphigus, Sjögren’s syndrome, and OLP [10–12]. OLL can be induced by several systemic diseases such as graft-versus-host disease, systemic lupus erythematosus and malignant tumors [3]. However, OLL as an early manifestation of CD is not commonly reported. An increasing number of studies have investigated the possible relationship between thyroid diseases (Hashimoto thyroiditis, hyperthyroidism, thyroid cancer, etc.) and OLP/OLL [13–15]. However, OLL coexisting with CD and papillary thyroid carcinoma (PTC) is very rarely described. Here we present a rare case of OLL with CD and PTC simultaneously. The purpose of this case is to increase the awareness of correct diagnosis and effective treatment of OLL especially with systemic diseases.

## Case presentation

**Fig. 1:**
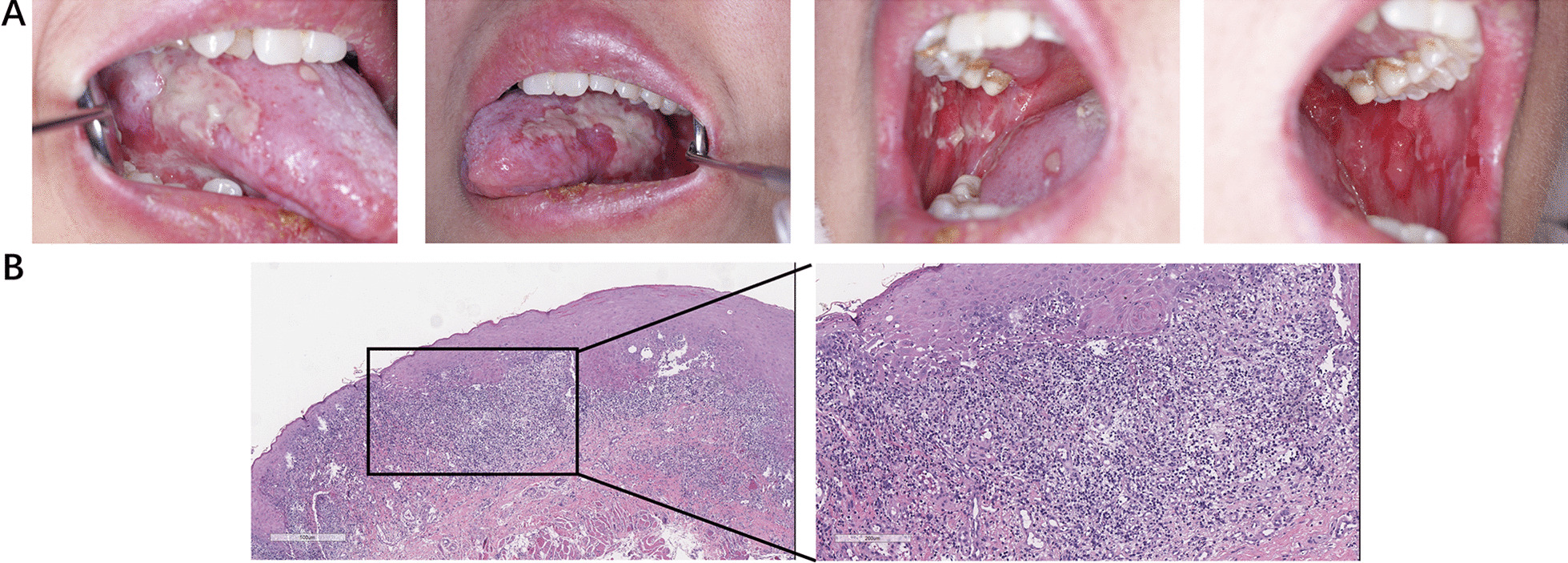
Clinical manifestations and histopathological features of the oral mucosa. **A** Clinical findings of the extensive lesions on the oral mucosa on the first visit to Hospital of Stomatology, Sun Yat-sen University in November 2021. **B** Histopathology revealed dyskeratosis of spinous cells, liquefaction and degeneration of basal cells, indistinct basement membrane, and a large number of lymphocytic infiltrates in all epidermal layers, which are consistent with oral lichenoid lesion changes. Scale bars, 500 µm for 40 × magnification and 200 µm for 100 × magnification

A 39-year-old female presented painful ulceration on the oral mucosa for the past two months and visited the Hospital of Stomatology, Sun Yat-sen University in November 2021. She had received treatment for this condition with a systemic administration of corticosteroids for more than one month in other hospital without efficacy. She denied any history of other systemic medications, dental restorations, radiotherapy, smoking and tobacco use. She had no family history of relevant conditions and malignancies. Physical examination revealed extensive ulcerated lesions on the tongue dorsum, bilateral tongue and buccal mucosa as well as the lower lip, and Nikolsky’s sign was negative (Fig. 1A). A biopsy from the left tongue showed dyskeratosis of spinous cells, liquefaction and degeneration of basal cells, indistinct basement membrane, and a large number of lymphocytic infiltrates in all epidermal layers instead of band-like infiltrates, which are consistent with pathological changes of OLL (Fig. 1B). Oral thalidomide and topical tacrolimus mouthwashes were scheduled to control the OLL, while the oral lesions remained unhealed. We recommended the patient for further systemic examination.

**Fig. 2:**
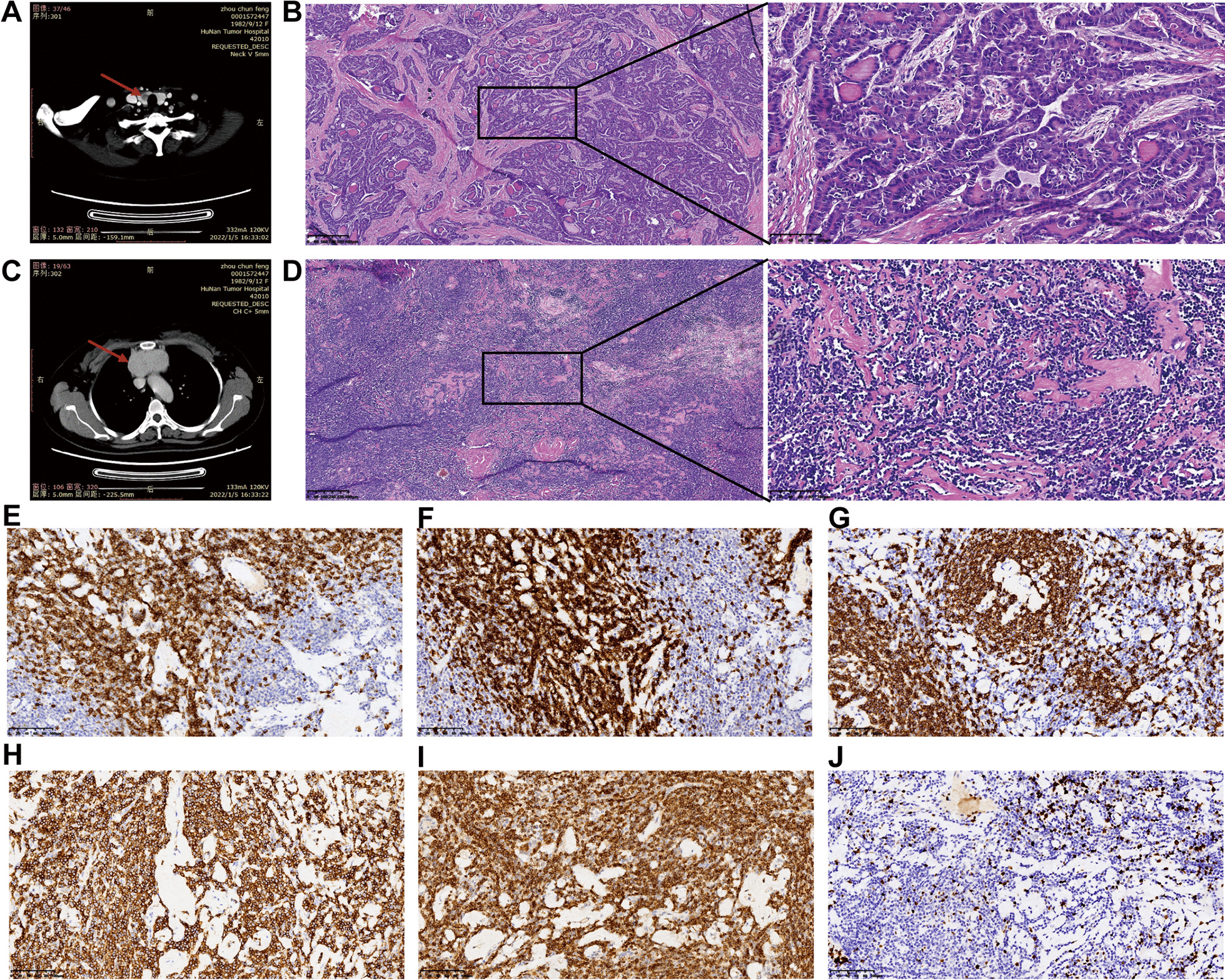
Contrast-enhanced computed tomography (CECT) and histopathological features of the thyroid and mediastinal masses. **A** CECT revealed a right low-density thyroid mass. **B** Histopathology from the right lobe of the thyroid confirmed papillary carcinoma. **C** CECT revealed a homogeneous soft-tissue mediastinal mass. **D** Histopathology from the mediastinal mass confirmed Castleman’s disease (unicentric, hyaline vascular type). Scale bars, 400 µm for 40 × magnification and 100 µm for 200 × magnification. **E**–**J** Immunohistochemical staining of the mediastinal sample for CD3 (**E**), CD5 (**F**), CD20 (**G**), CD45 (**H**), Bcl-2 (**I**), Ki67 (**J**). Scale bars, 100 µm for 200 × magnification

In January 2022, the patient found a goiter in the right neck, accompanied with extensive unhealed ulcerated lesions on the oral mucosa. The patient visited Xiangya Hospital, Central South University for further examination. Contrast-enhanced computed tomography (CECT) confirmed the thyroid mass and revealed a mediastinal mass unexpectedly (Fig. 2A, C). Laboratory examination showed procalcitonin (PCT), C-reactive protein (CRP), erythrocyte sedimentation rate (ESR), interleukin-1β (IL-1β) and cancer antigen 125 (CA125) were obviously elevated (PCT: 0.104 ng/mL, normal: < 0.06 ng/mL; CRP: 14.70 mg/L, normal: 0–8.00 mg/L; ESR: 46.00 mm/h, normal: 0–26.00 mm/h; IL-1β: 7.11 pg/mL, normal: < 5 pg/mL; CA125: 76.6 U/mL, normal: 0–35 U/mL). The neck and mediastinal masses were surgically removed and the histopathologic examination showed PTC in the right lobe of the thyroid (Fig. 2B) and CD (unicentric, hyaline vascular type) in the anterior mediastinal mass (Fig. 2D). Further immunohistochemical staining confirmed the diagnosis of CD (Fig. 2E-–J). After thyroid cancer surgery, oral levothyroxine was scheduled for the inhibition and replacement therapy, and topical tacrolimus mouthwashes were applied to subdue pain and inflammation for oral lesions.

**Fig. 3:**
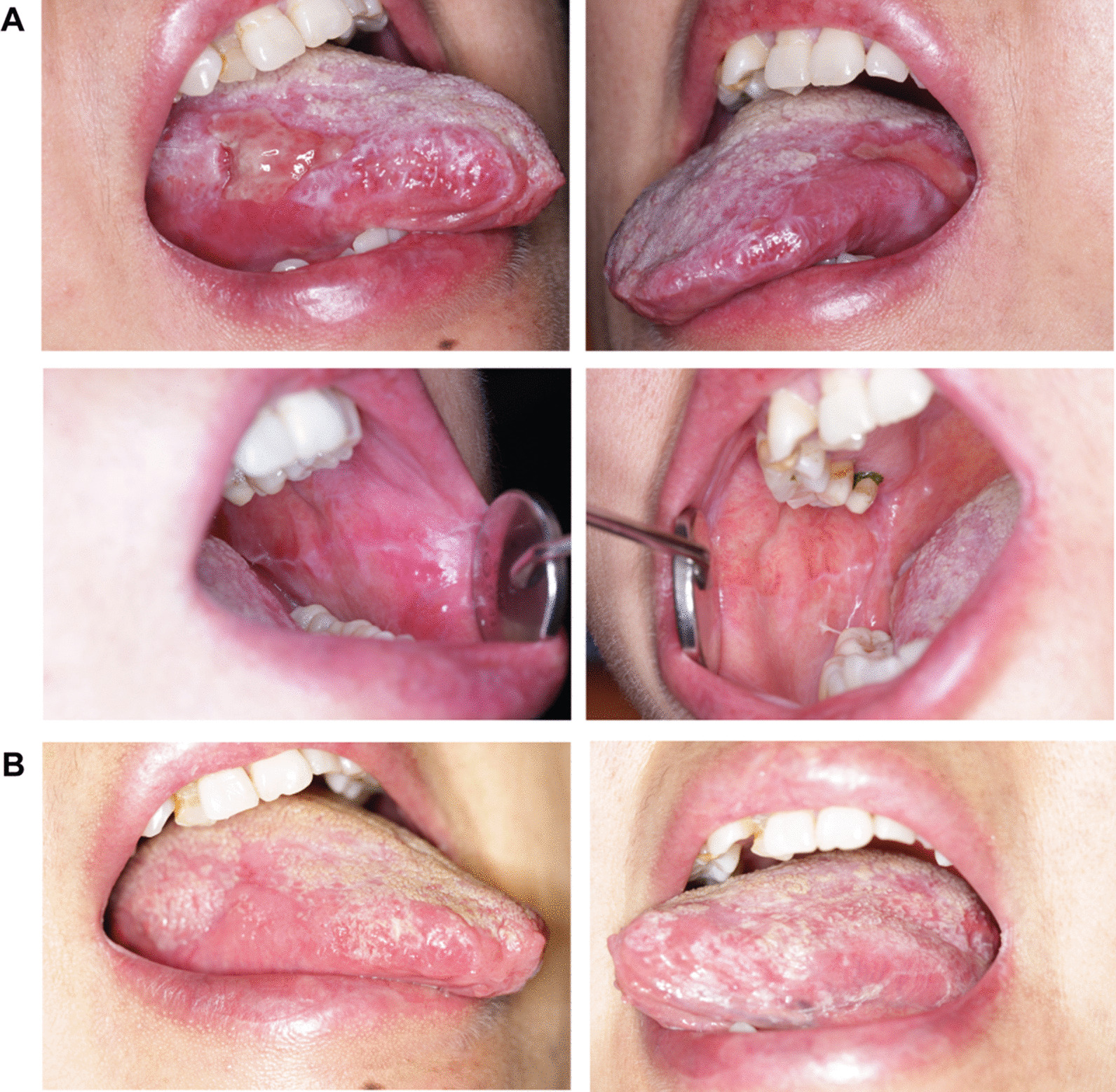
Clinical manifestations of the oral mucosa after the excision of neoplasms. **A** Ulcerated lesions on the bilateral tongue significantly alleviated but remained unhealed. Lesions on the buccal mucosa were healed in March 2022. **B** Healed lesions on the oral mucosa after multidisciplinary treatment in April 2022

**Fig. 4:**
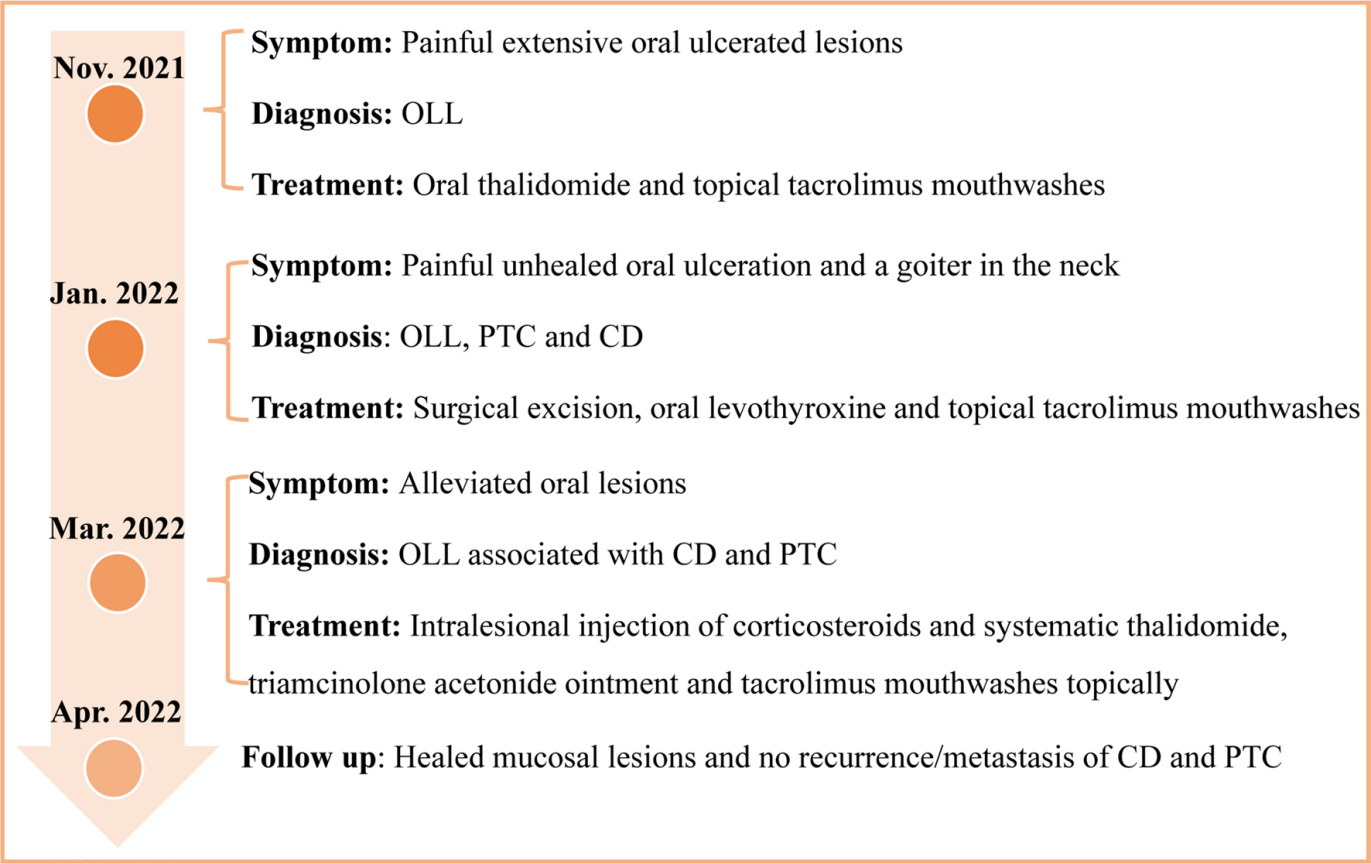
The management of the patient. OLL Oral lichenoid lesion; PTC Papillary thyroid carcinoma; CD Castleman’s disease

In March 2022, two months after complete excision of the neoplasms, the patient revisited to the Hospital of Stomatology, Sun Yat-sen University. The mucosal involvement showed significant alleviation, but there remained unhealed ulceration on the bilateral tongue (Fig. 3A). Based on the manifestations and findings across the timeline, a definitive diagnosis of OLL associated with CD and PTC was made. We subsequently gave the patient intralesional injection of corticosteroids and systematic thalidomide, and applied triamcinolone acetonide ointment and tacrolimus mouthwashes topically. After one month, the tongue mucosal lesions gradually healed (Fig. 3B). Positron emission tomography/computed tomography confirmed that there was no recurrence/metastasis of CD and PTC. The management of the patient was summarized in Fig. 4.

We followed the guidelines of the Helsinki Declaration in this investigation. The procedure has been explained to the patient, who signed an informed consent allowing treatment procedures and publication of her data.

## Discussion and conclusion

OLL has been classified as an independent disease entity in oral potential malignant disorders (OPMDs), and presents a higher risk of malignant transformation compared with OLP [16, 17]. Diagnosis of OLL has been a challenging endeavor due to the presence of overlapping features and inflammatory infiltration with OLP [18]. The definitive diagnosis must be based on patient’s history, clinical and histopathological characteristics [19]. Currently, there is no precise cure for OLL. OLL and OLP share similar therapeutic regimens to alleviate symptoms, lower the inflammatory response, heal ulcerated lesions and decrease the risk of malignant transformation [5, 20]. Topical corticosteroids may be the first line of treatment for symptomatic OLL. When topical therapy proves ineffective and the symptoms are stubborn, systemic corticosteroids are recommended [4, 20]. Nevertheless, for our patient, because of the persistence of the thyroid and mediastinal neoplasms, systemic corticosteroids treatment was ineffective. The combination of systemic thalidomide and topical corticosteroids/tacrolimus may be considered as an alternative intervention in OLL patients [5].

The oral cavity can be regarded as the mirror of systemic health, since many systemic diseases may have manifestations on the oral mucosa [21]. In this case, OLL was a mucosal complication of CD and PTC. A retrospective study has shown that 47% cases of CD were accompanied with cutaneous/mucosal lichen planus-like lesions [12]. The underlying mechanism is not fully understood, which may be related to the autoantibody production, epitope spreading, antigen mimicry and interleukin-6 upregulation [22, 23]. Nonetheless, to our knowledge, no study has reported a definite OLL case associated with CD. It has been reported OLL can be associated with thyroid diseases [6, 13]. A retrospective analysis found that 14.1% OLL patients had thyroid diseases [6]. One of the hypotheses for the connection between OLL and thyroid diseases is the presence of a common autoimmune process [24]. Robledo-Sierra et al. proposed a specific subgroup of patients with both oral lichenoid disease and thyroid disorders [13]. Nevertheless, OLL accompanied with CD and PTC simultaneously has not been reported. Hence, it is important to scrutinize the predisposing factors of OLL for better treatment.

Before her first visit to our department, she had received oral corticosteroid treatment for more than one month in other hospital without efficacy and suffered from refractory OLL. After initial systemic treatment with thalidomide for oral ulceration, the oral lesions still persisted. Therefore, in addition to timely adjusting the treatment regimen, the elimination of potential provoking factors is an important step in the management of OLL. After the excision of thyroid and mediastinal neoplasms, the oral lesions gradually got remission. With subsequent treatment for stubborn ulcerated lesions, the patient’s OLL showed an obvious healing. Hence, clinicians should pay attention to the treatment of systemic diseases as well as oral lesions.

Collectively, we present a rare OLL case with CD and PTC simultaneously. This case reminds us to focus on the identification of underlying etiologies, especially for patients with systemic diseases. Multidisciplinary collaboration is needed to diagnose and treat such patients. Furthermore, with respect to the malignant potential, OLL should be regularly monitored as well as systemically treated.

## Data Availability

The datasets generated and analyzed during the current study are not publicly available since they contain medical information of the patient.

## Abbreviations

OLL: Oral lichenoid lesion
OLP: Oral lichen planus
CD: Castleman’s disease
PTC: Papillary thyroid carcinoma
CECT: Contrast-enhanced computed tomography
PCT: Procalcitonin
CRP: C-reactive protein
ESR: Erythrocyte sedimentation rate
IL-1β: Interleukin-1β
CA125: Cancer antigen 125
OPMDs: Oral potential malignant disorders
